# Exploring drug cost and disease outcome in rheumatoid arthritis patients treated with biologic and targeted synthetic DMARDs in Norway in 2010–2019 – a country with a national tender system for prescription of costly drugs

**DOI:** 10.1186/s12913-021-07425-w

**Published:** 2022-01-10

**Authors:** Alen Brkic, Andreas P. Diamantopoulos, Espen Andre Haavardsholm, Bjørg Tilde Svanes Fevang, Lene Kristin Brekke, Liz Loli, Camilla Zettel, Erik Rødevand, Gunnstein Bakland, Pawel Mielnik, Glenn Haugeberg

**Affiliations:** 1 Research Department, Sorlandet Hospital, Kristiansand, Norway; 2 Department of Rheumatology, Martina Hansens Hospital, Bærum, Oslo, Norway; 3 Division of Rheumatology and Research, Diakonhjemmet Hospital, Oslo, Norway; 4 Institute of Clinical Medicine, University of Oslo, Oslo, Norway; 5 Bergen Group of Epidemiology and Biomarkers in Rheumatic Disease, Department of Rheumatology, Haukeland University Hospital, Bergen, Norway; 6 Haugesund Hospital for Rheumatic Diseases, Haugesund, Norway; 7 Lillehammer Hospital for Rheumatic Diseases, Lillehammer, Norway; 8 Department of Rheumatology, Betanien Hospital, Skien, Norway; 9 Department of Rheumatology, St Olavs Hospital, Trondheim University Hospital, Trondheim, Norway; 10 Department of Rheumatology, University Hospital of North Norway, Tromsø, Norway; 11 Section for Rheumatology, Department for Neurology, Rheumatology and Physical Medicine, District General Hospital of Førde, Førde, Norway; 12 Division of Rheumatology, Department of Medicine, Sorlandet Hospital, Kristiansand, Norway; 13 Department of Neuromedicine and Movement Science, Faculty of Medicine and Health Sciences, NTNU, Norwegian University of Science and Technology, Trondheim, Norway

**Keywords:** Rheumatoid arthritis, Economics, Biological therapy, Biosimilar pharmaceuticals

## Abstract

**Background:**

In Norway, an annual tender system for the prescription of biologic and targeted synthetic disease-modifying antirheumatic drugs (b/tsDMARDs) has been used since 2007. This study aimed to explore annual b/tsDMARDs costs and disease outcomes in Norwegian rheumatoid arthritis (RA) patients between 2010 and 2019 under the influence of the tender system.

**Methods:**

RA patients monitored in ordinary clinical practice were recruited from 10 Norwegian centers. Data files from each center for each year were collected to explore demographics, disease outcomes, and the prescribed treatment. The cost of b/tsDMARDs was calculated based on the drug price given in the annual tender process.

**Results:**

The number of registered RA patients increased from 4909 in 2010 to 9335 in 2019. The percentage of patients receiving a b/tsDMARD was 39% in 2010 and 45% in 2019. The proportion of b/tsDMARDs treated patients achieving DAS28 remission increased from 42 to 67%. The estimated mean annual cost to treat a patient on b/tsDMARDs fell by 47%, from 13.1 thousand euros (EUR) in 2010 to 6.9 thousand EUR in 2019. The mean annual cost to treat b/tsDMARDs naïve patients was reduced by 75% (13.0 thousand EUR in 2010 and 3.2 thousand EUR in 2019).

**Conclusions:**

In the period 2010–2019, b/tsDMARD treatment costs for Norwegian RA patients were significantly reduced, whereas DAS28 remission rates increased. Our data may indicate that the health authorities’ intention to reduce treatment costs by implementing a tender system has been successful.

**Supplementary Information:**

The online version contains supplementary material available at 10.1186/s12913-021-07425-w.

## Background

The introduction of biologic and targeted synthetic disease-modifying antirheumatic drugs (b/tsDMARDs), early intervention, and treat to target strategies represents a paradigm shift in the treatment of patients with inflammatory joint disorders, e.g., rheumatoid arthritis (RA), where remission is now an attainable treatment goal [1–4]. However, the high cost of b/tsDMARDs has caused restrictions on the usage of these drugs, contributing to inequality of care worldwide [5–7].

In some countries (e.g., Norway and Denmark) with a public tax-funded healthcare system, tender systems, and the possibility of a mandatory switch to potentially cheaper biosimilar drugs have been implemented to reduce the drug expenditure (particularly for costly drugs). To our knowledge, this is the first study to explore changes in b/tsDMARD treatment costs set against changes in disease outcomes in RA following the implementation of a tender system. This study aimed to explore treatment cost and disease outcomes in RA patients treated with b/tsDMARDs in Norway during a 10-year period (2010 to 2019) with a tender system in effect.

## Methods

### Patient inclusion and data collection

Data were obtained from the BioRheuma project (BIOlogic treatment of patients suffering from inflammatory RHEUMAtic disorders in Norway) that started in 2010. The objective of the BioRheuma project was to facilitate the use of recommended and validated outcome measures to monitor patients with inflammatory joint disorders as part of ordinary care in Norwegian outpatient clinics. Patient monitoring at the participating centers was standardized using the computer tool GoTreatIT® Rheuma (www.diagraphit.com). The clinical expectations of the project were to reveal annual changes in the usage of conventional synthetic DMARDs (csDMARDs) and b/tsDMARDs, viewed against changes in demographics, disease activity, and patient-reported outcome measures (PROMs) during follow-up.

The 10 BioRheuma centers providing data for this study were located across the country (Bergen, Bærum, Førde, Haugesund, Kristiansand, Lillehammer, Oslo, Skien, Tromsø, and Trondheim). We estimated the completeness of included patients from each center by comparing with published prevalence figures for RA in Norway [8, 9]. BioRheuma prevalence figures were calculated using the number of included RA patients at each center divided by the background population the various centers were covering.

For each of the 10 years, data was extracted from each participating center’s database using predefined queries. One query retrieved RA patients registered with at least one visit in the examined year. Data from the latest visit was used if multiple visits occurred in that year. Another query retrieved all patients starting on either bDMARD or tsDMARD for the different years. Anonymized data files from the 10 participating centers were merged and analyzed using EXCEL and the Statistical package for social sciences (SPSS).

Data collection for each year included demographic variables, diagnosis-related variables, disease activity measures, PROMs, and RA treatment medications. Demographic variables include patient age, sex, body mass index (BMI, kg/m2), current smoking status, years of education, disease duration, and occupational status. The occupational status of participants younger than 65 years was categorized as enabled workers or disabled workers. Patients who reported their occupational status as a full-time job, part-time job, student, maternity leave, paternity leave, sick leave, unemployed, early retirement, part-time job/sick leave, part-time job/unemployed were defined as “enabled workers.” In contrast, patients who reported part-time job/disabled pensioner, disabled pensioner, disabled pensioner due to RA, medical rehabilitation, and occupational rehabilitation were defined as “disabled workers.” Participants ≥ 65 years were omitted and defined as pensioners. Disease duration was calculated from the date of diagnosis until the latest visit at the outpatient clinic for the examined year.

Diagnosis-related variables include rheumatoid factor (RF) and anti-cyclic citrullinated peptide (anti-CCP). Measures reflecting disease activity encompass laboratory measures (erythrocyte sedimentation rate (ESR), C-reactive protein (CRP), the clinical measures 28 swollen and tender joint count (28SJC/28TJC), investigator global assessment (IGA) scored on a visual analog scale (VAS; 0–100 mm), and composite 28 joint count Disease Activity Score using CRP (DAS28) [10]. The PROMs included were pain, patient global assessment (PGA), and fatigue scored on a VAS-scale (0–100 mm), as well as morning stiffness (reported in 15-min units) and Modified Health Assessment Questionnaire (MHAQ) [11] to evaluate the physical function of the RA patients.

Among available composite scores, DAS28 was used to define the disease activity status with the following cut-off values; remission ≤2.6, low disease activity between > 2.6 and ≤ 3.2, moderate disease between > 3.2 and ≤ 5.1, and high disease activity for those > 5.1 [10].

### Drug costs analysis

For each of the 10 years, the annual total cost for b/tsDMARDs as well as mean b/tsDMARD cost per patient was calculated for all patients receiving ongoing b/tsDMARDs (current b/tsDMARD users), for those who started on their first b/tsDMARD (naïve b/tsDMARD users) and for those who started on a new b/tsDMARDs but were previous users of b/tsDMARDs. The cost was calculated based on price offers given for the separate drugs at the annual tender process for the given year. Adjusted cost was also calculated using the Norwegian consumer price index (CPI) for pharmaceuticals from 2010 Norwegian Kroners (NOK) [12]. Only average prices (no drug-specific prices) are presented due to an agreement between the pharmaceutical companies and the Norwegian authorities to keep the costs for individual drugs confidential and exempt from the public. Due to the challenging COVID-19 pandemic situation, clinical data for 2020 was not collected, but the cost for 2020 was calculated using 2019 population data. All costs were converted to euros (EUR) based on the average NOK-to-EUR conversion rate between 2010 and 2020 (1 NOK = 8.839 EUR).

The b/tsDMARDs included were Tumor Necrosis Factor inhibitors (TNFi) (etanercept reference, etanercept SB4, infliximab reference, infliximab CT-P13, adalimumab, golimumab, certolizumab pegol), non-TNFi (rituximab reference, rituximab GP2013, abatacept, and tocilizumab), and tsDMARDs (baricitinib and tofacitinib). For 2020 the biosimilars infliximab GP1111 and adalimumab GP2017 won the tender and were used in the cost analysis for 2020. Data collection also included the use of csDMARDs and prednisolone**.**

### Statistical analysis

Categorical variables are reported as numbers and percentages and continuous variables as mean with standard deviation (SD), or mean with range. Change and association between variables over the 10-year period were analyzed with SPSS using one-way analysis of variance (ANOVA) for continuous variables and the chi-square test for categorical variables. Only available data were used without imputation of missing data. A *p*-value of < 0.05 was considered statistically significant.

### Ethics

The study was approved by the regional ethical committee (REC) (Regional etisk komite Midt-Norge 2010/3078) and follows the Declaration of Helsinki ethical principles of medical research involving human subjects. No consent from patients was required by the REC, as all data were anonymized and collected as part of routine clinical care.

## Results

### Demographics, disease activity, and patient-reported outcomes

The number of RA patients registered in the BioRheuma project in the 10-year period ranged from 4909 patients in 2010 to a maximum of 9335 in 2019, and the percentage of patients registered as b/tsDMARD users increased from 40% (*n* = 1959) to 45% (*n* = 4209), respectively. In Table 1, annual results are shown for demographics, biomarkers, disease activity, and PROM variables for current users of b/tsDMARDs. The percentage of patients currently treated with b/tsDMARDs increased from 39% in 2010 to 45% in 2019. An improvement was seen for disease activity measures, MHAQ, and fatigue, but not for PGA, pain, and morning stiffness. The proportion of patients in DAS28 remission who received a b/tsDMARD increased from 42% in 2010 to 67% in 2019. The percentage of enabled workers did not change significantly, ranging from 63% in 2010 to 59% in 2019.

**Table 1:** Demographic and disease characteristics in Norwegian RA patients currently using b/tsDMARDs during 2010–2019

Ten year period	2010	2011	2012	2013	2014	2015	2016	2017	2018	2019	Mean	Missing Data Mean, Range	***P***-value
**The annual number of patients**													
BioRheuma patients, N	4909	7256	7993	7278	8023	9057	9176	9225	9102	9335			
b/tsDMARDs users, N (%)	1936 (39)	2855 (39)	3136 (39)	3060 (42)	3419 (43)	3688 (41)	3770 (41)	3869 (42)	3869 (43)	4154 (45)	41%		
**Demographics**													
Age (years)	60 (13)	60 (14)	59 (14)	59 (14)	59 (14)	59 (14)	59 (14)	59 (14)	60 (14)	60 (14)	59.3	1.1%, 0–11%	0.044
Female	74%	73%	72%	72%	73%	73%	73%	73%	73%	72%	73%	1.1%, 0–11%	0.938
BMI (kg/m2)	26 (4.9)	26 (4.6)	26 (4.6)	26 (4.6)	26 (4.6)	26 (4.7)	26 (4.8)	26 (4.8)	26 (4.6)	26 (4.9)	26	9.9%, 2–57%	< 0.001
Education (years)	13 (3.6)	12 (3.8)	12 (3.7)	12 (3.7)	12 (3.7)	12 (3.7)	12 (3.7)	13 (3.7)	13 (3.7)	13 (3.7)	12	9.3%, 1–55%	< 0.001
Current Smokers	23%	22%	20%	19%	18%	17%	16%	15%	14%	14%	18%	8.2%, 1–50%	< 0.001
Disease Duration (years)	13 (10)	13 (11)	14 (11)	14 (11)	14 (11)	14 (11)	15 (11)	14 (11)	15 (11)	15 (11)	14	0.0%, 0–0%	< 0.001
Enabled Workers	63%	59%	59%	60%	57%	58%	59%	59%	59%	59%	59%	7.4%, 1–45%	0.393
**Biomarkers**													
CCP Positive	82%	82%	81%	81%	80%	80%	81%	81%	81%	81%	81%	27%, 14–42%	0.900
RF Positive	75%	75%	74%	73%	73%	73%	72%	73%	73%	72%	73%	45%, 29–61%	0.798
**Disease Activity**													
ESR (mm/h)	19 (16) [14 (17)]	18 (16) [13 (16)]	16 (15) [12 (15)]	16 (14) [12 (14)]	15 (15) [11 (14)]	15 (15) [11 (14)]	14 (14) [10 (13)]	14 (14) [9 (13)]	14 (15) [9 (13)]	14 (15) [9 (12)]	16	26%, 20–32%	< 0.001
CRP (mg/L)	8.4 (17) [4 (7)]	7.8 (14) [4 (7)]	6.6 (12) [3 (5)]	6.2 (10) [3 (5)]	6.2 (11) [3 (5)]	6.4 (13) [3 (6)]	6.4 (17) [3 (4)]	5.7 (11) [2 (4)]	5.9 (11) [2 (4)]	6.0 (11) [2 (4)]	6.6	19%, 15–27%	< 0.001
TJC28 (0–28)	3.4 (4.7) [2 (5)]	3.1 (4.5) [1 (4)]	2.7 (4.4) [1 (3)]	2.5 (4.1) [1 (3)]	2.4 (3.9) [1 (3)]	2.1 (3.7) [1 (3)]	2.1 (3.7) [1 (2)]	1.9 (3.7) [0 (2)]	1.9 (3.6) [0 (2)]	1.7 (3.4) [0 (2)]	2.4	15%, 9–19%	< 0.001
SJC28 (0–28)	2.3 (3.4) [1 (3)]	2.0 (3.1) [1 (3)]	1.9 (3.1) [1 (2)]	1.5 (2.6) [0 (2)]	1.2 (2.3) [0 (2)]	1.1 (2.2) [0 (1)]	1.1 (2.1) [0 (1)]	1.1 (2.2) [0 (1)]	0.9 (2.0) [0 (1)]	0.8 (2.0) [0 (1)]	1.4	15%, 9–19%	< 0.001
IGA (VAS, 0–100 mm)	18 (16)	18 (16)	17 (16)	16 (15)	16 (15)	15 (15)	14 (15)	14 (15)	13 (15)	12 (14)	15.2	40%, 35–50%	< 0.001
DAS28(4)-CRP	3.1 (1.2)	3.0 (1.2)	2.8 (1.2)	2.7 (1.1)	2.6 (1.1)	2.6 (1.1)	2.6 (1.1)	2.5 (1.1)	2.5 (1.1)	2.4 (1.1)	2.7	30%, 26–34%	< 0.001
DAS28 Remission	42%	46%	51%	56%	57%	59%	60%	63%	64%	67%	56%	30%, 26–34%	< 0.001
DAS28 LDA	18%	17%	17%	17%	16%	16%	16%	15%	16%	14%	16%	30%, 26–34%	0.034
**Patient-Reported Outcome Measures**													
PGA (VAS, 0–100 mm)	33 (24)	33 (25)	33 (25)	32 (25)	32 (25)	33 (25)	33 (25)	32 (26)	32 (26)	32 (26)	33	10%, 8–11%	0.270
Pain (VAS, 0–100 mm)	32 (25)	34 (25)	33 (24)	32 (25)	32 (25)	33 (25)	32 (25)	32 (26)	32 (25)	32 (26)	33	20%, 14–53%	0.204
MHAQ (0–3)	0.5 (0.5)	0.5 (0.5)	0.5 (0.5)	0.5 (0.5)	0.5 (0.5)	0.5 (0.5)	0.5 (0.5)	0.5 (0.5)	0.5 (0.5)	0.5 (0.5)	0.5	15%, 10–37%	0.001
Fatigue (VAS, 0–100 mm)	38 (29)	37 (29)	38 (29)	37 (29)	38 (30)	39 (30)	39 (30)	39 (30)	40 (310)	40 (31)	39	37%, 15–54%	0.006
Morning Stiffness (hr)	0.9 (1.2)	0.9 (1.3)	0.9 (1.2)	0.9 (1.2)	0.8 (1.2)	0.9 (1.2)	0.9 (1.3)	0.9 (1.2)	0.9 (1.2)	0.9 (1.2)	0.9	42%, 16–59%	0.614

*Note*: Categorical variables are presented as percentages and continuous variables as mean with standard deviation (SD). Variables ESR, CRP, TJC28, and SJC28 also show median with interquartile range [Median (Interquartile Range)] below their Mean (SD). Missing data are presented as mean with range. χ^2^ test for categorical variables and one-way ANOVA for continuous variables was used to test for differences during follow-up of ten years

Occupation Status: Enabled Workers (< 65 years old) = Full Job, Part-time Job, Student, Maternity Leave, Paternity leave, Sick Leave, Unemployed, Early Retirement, Part-time job/Sick Leave, Part-time job/Unemployed), Disabled Workers (< 65 years) = Part-time Job/Disabled Early Retirement, Early Retirement due to Disability, Early Retirement due to RA, Medical Rehabilitation, Occupational Rehabilitation

*Abbreviations*: *RA* Rheumatoid Arthritis, *b/tsDMARDs* biologic and target synthetic Disease-Modifying Antirheumatic Drugs, *BMI* Body Mass Index, *CCP* Anti-cyclic citrullinated peptide, *RF* Rheumatoid Factor, *ESR* Erythrocyte Sedimentation Rate, *CRP* C-Reactive Protein, *TJC28* Tender 28-Joint Count, *SJC28* Swollen 28-Joint Count, *IGA* Investigators Global Assessment, *VAS* Visual Analog Scale (Measured 0–100), *DAS28* Disease Activity Score, *LDA* Low Disease Activity, *PGA* Patient Global assessment, *MHAQ* Modified Health Assessment Questionnaire

A supplementary table (see Additional file 1) compares mean values and range for the 10 years between b/tsDMARD-treated patients and non-b/tsDMARDs RA patients. In general, no relevant differences for disease activity measures and PROMs were seen between b/tsDMARDs and non-b/tsDMARDs treated RA patients. However, more b/tsDMARDs treated patients were RF and CCP positive. Numerically only minor, yet statistically significant differences were found for most demographic variables. However, disease duration was markedly longer for b/tsDMARDs than non-b/tsDMARDs treated patients (14.0 vs. 8.9 years, *p* = < 0.001).

Baseline values for demographics, disease activity, and PROMs are shown in Table 2 for naïve b/tsDMARDs users and in Table 3 for patients starting subsequent b/tsDMARD. For patients naïve to b/tsDMARDs, disease duration was the only demographic variable with a significant change during the 10 years. In contrast, significant changes were found for all demographic variables apart from work status in the non-naïve group.

**Table 2:** Demographic and disease characteristics in Norwegian RA patients starting naïve on b/tsDMARDs during 2010–2019

Ten year period	2010	2011	2012	2013	2014	2015	2016	2017	2018	2019	Mean	Missing Data Mean, Range	***P***-value
**The annual number of patients**													
All b/tsDMARDs starters	832	887	875	857	852	946	1671	1342	1068	1475			
Naïve starters, N (%)	378 (45%)	424 (48%)	421 (48%)	386 (45%)	356 (42%)	367 (39%)	400 (24%)	418 (31%)	408 (38%)	409 (28%)	39%		
**Demographics**													
Age (years)	57 (15)	56 (15)	57 (14)	55 (15)	55 (15)	55 (14)	56 (15)	54 (16)	55 (15)	55 (15)	55	0.3%, 0–2%	0.212
Female	71%	76%	68%	71%	69%	71%	75%	69%	69%	70%	71%	0.1%, 0–1%	0.152
BMI (kg/m2)	25 (4.3)	26 (4.3)	26 (4.7)	26 (4.5)	26 (4.7)	26.1 (5.1)	26 (4.5)	26 (5.1)	27 (5.3)	26 (5.3)	26	23%, 11–66%	0.256
Education (years)	13 (3.7)	12 (3.6)	13 (3.6)	12 (3.9)	13 (3.7)	13 (3.7)	13 (3.9)	12 (3.5)	13 (3.8)	13 (3.7)	13	25%, 18–64%	0.683
Current Smokers	25%	22%	20%	17%	15%	17%	20%	16%	17%	18%	19%	24%, 17–64%	0.175
Disease Duration	4.6 (5.6)	7.2 (9.3)	7.8 (9.9)	5.9 (7.9)	6.4 (8.5)	6.4 (9.0)	7.1 (9.4)	5.8 (8.9)	4.9 (7.2)	5.2 (7.0)	6.1	28%, 0–78%	< 0.001
Enabled Workers	71%	73%	73%	76%	74%	70%	73%	72%	69%	73%	72%	24%, 16–63%	0.922
**Disease Activity**													
ESR (mm/h)	26 (22) [20 (24)]	26 (22) [20 (23)]	22.6 (20) [16 (21)]	22 (18) [16 (19)]	22.1 (18) [19 (20)]	23 (19) [17 (22)]	22(21) [15 (21)]	22 (18) [17 (21)]	20 (19) [13 (18)]	20 (18) [14 (18)]	23	23%, 15–30%	< 0.001
CRP (mg/L)	17 (24) [8 (16)]	16.9 (29) [7 (16)]	14.2 (20) [7 (14)]	14 (21) [7 (12)]	15.2 (22) [6 (14)]	11 (14) [6 (11)]	15 (26) [5 (13)]	12.9 (16) [6 (17)]	11 (17) [5 (10)]	12 (18) [5 (13)]	14	16%, 8–26%	< 0.001
TJC28 (0–28)	8.3 (6.8) [7 (8)]	7.4 (6.6) [5 (9)]	6.5 (5.9) [5 (8)]	5.8 (5.3) [5 (6)]	6.7 (6.0) [5 (8)]	6.4 (5.8) [5 (7)]	5.6 (5.5) [4 (7)]	6.2 (6.2) [4 (7)]	5.3 (5.4) [4 (6)]	5.1 (5.6) [3 (7)]	6.3	13%, 7–21%	< 0.001
SJC28 (0–28)	6.8 (5.0) [6 (6)]	6.0 (4.9) [5 (6)]	5.4 (4.5) [4 (6)]	4.8 (4.2) [4 (5)]	4.9 (4.5) [4 (6)]	4.8 (4.9) [3 (6)]	4.1 (4.4) [3 (5)]	4.0 (3.9) [3 (5)]	4.0 (4.5) [3 (5)]	3.5 (4.2) [2 (6)]	4.8	13%, 7–21%	< 0.001
IGA (VAS, 0–100 mm)	39 (18)	40.2 (20)	39.1 (19)	38 (18)	38 (18)	37 (18)	38 (20)	36 (18)	33 (19)	32 (20)	37	32%, 19–45%	< 0.001
DAS28	5.0 (1.4)	4.6 (1.3)	4.5 (1.3)	4.3 (1.3)	4.5 (1.4)	4.4 (1.5)	4.3 (1.5)	4.3 (1.5)	4.1 (1.4)	3.8 (1.4)	4.4	29%, 19–35%	< 0.001
**Patient-Reported Outcome Measures**													
PGA (VAS, 0–100 mm)	54 (25)	50 (25)	51 (26)	48 (25)	53 (26)	49 (26)	50 (26)	50 (26)	49 (26)	47 (26)	50	13%, 8–21%	0.014
Pain (VAS, 0–100 mm)	51 (27)	46 (25)	49 (25)	47 (25)	48 (25)	47 (24)	47 (27)	47 (25)	45 (26)	45 (27)	47	22%, 10–57%	0.357
MHAQ (0–3)	0.7 (0.6)	0.7 (0.5)	0.7 (0.5)	0.6 (0.5)	0.7 (0.5)	0.6 (0.5)	0.6 (0.5)	0.7 (0.5)	0.6 (0.5)	0.6 (0.5)	0.6	18%, 9–39%	0.007
Fatigue (VAS, 0–100 mm)	50 (29)	48 (30)	49 (29)	46 (30)	51 (30)	50 (30)	46 (31)	49 (30)	48 (30)	48 (33)	49	36%, 17–51%	0.725
Morning Stiffness (hr)	1.7 (1.5)	1.6 (1.6)	1.6 (1.5)	1.6 (1.6)	1.8 (1.8)	1.5 (1.5)	1.5 (1.6)	1.7 (1.5)	1.3 (1.5)	1.4 (1.4)	1.6	38%, 17–53%	0.040

*Note*: Categorical variables are presented as percentages and continuous variables as mean with standard deviation (SD). Variables ESR, CRP, TJC28, and SJC28 also show median with interquartile range [Median (Interquartile Range)] below their Mean (SD). Missing data are presented as mean with range. χ^2^ test for categorical variables and one-way ANOVA for continuous variables was used to test for differences during follow-up of ten years

Occupation Status: Enabled Workers (< 65 years old) = Full Job, Part-time Job, Student, Maternity Leave, Paternity leave, Sick Leave, Unemployed, Early Retirement, Part-time job/Sick Leave, Part-time job/Unemployed), Disabled Workers (< 65 years) = Part-time Job/Disabled Early Retirement, Early Retirement due to Disability, Early Retirement due to RA, Medical Rehabilitation, Occupational Rehabilitation

*Abbreviations*: *RA* Rheumatoid Arthritis, *b/tsDMARDs* biologic and target synthetic Disease-Modifying Antirheumatic Drugs, *BMI* Body Mass Index, *CCP* Anti-cyclic citrullinated peptide, *RF* Rheumatoid Factor, *ESR* Erythrocyte Sedimentation Rate, *CRP* C-Reactive Protein, *TJC28* Tender 28-Joint Count, *SJC28* Swollen 28-Joint Count, *IGA* Investigators Global Assessment, *VAS* Visual Analog Scale (Measured 0–100), *DAS28* Disease Activity Score, *LDA* Low Disease Activity, *PGA* Patient Global assessment, *MHAQ* Modified Health Assessment Questionnaire

**Table 3:** Demographic and disease characteristics in Norwegian RA patients starting non-naïve on b/tsDMARDs during 2010–2019

Ten year period	2010	2011	2012	2013	2014	2015	2016	2017	2018	2019	Mean	Missing Data Mean, Range	***P*** value
**The annual number of patients**													
All b/tsDMARDs starters	832	887	875	857	852	946	1671	1342	1068	1475			
Non-naïve starters, N (%)	454 (55)	463 (52)	454 (52)	471 (55)	496 (58)	579 (61)	1271 (76)	924 (69)	660 (62)	1066 (72)	61%		
**Demographics**													
Age (years)	59 (14)	57 (14)	58 (14)	57 (15)	55 (14)	578 (15)	59 (14)	58 (14)	58 (15)	59 (14)	58	0.3%, 0–3%	< 0.001
Female	75%	79%	78%	75%	790%	79%	71%	76%	75%	74%	76%	0.0%, 0–0%	0.002
BMI (kg/m2)	26 (5.5)	25 (4.6)	26 (4.8)	26 (5.1)	26 (4.4)	26 (5.1)	26 (4.7)	26 (4.7)	26 (5.1)	27 (5.0)	26	19%, 6–65%	< 0.001
Education (years)	12 (3.4)	12 (3.9)	12 (3.6)	12 (3.8)	13 (4.0)	12 (3.7)	12 (3.6)	13 (3.8)	13 (3.8)	13 (3.7)	13	29%, 18–59%	0.019
Current Smokers	27%	26%	22.0%	17%	14%	19%	18%	16%	12%	14%	18%	28%, 18–58%	< 0.001
Disease Duration	11 (10)	11 (9.8)	10 (8.6)	13 (11)	12 (9.7)	13 (10)	14 (11)	13 (10)	13 (11)	13 (11)	12	28%, 0–81%	0.001
Enabled Workers	54%	51%	52%	63%	57%	54%	60%	59%	59%	58%	57%	26%, 15–54%	0.106
**Disease Activity**													
ESR (mm/h)	32 (24) [25 (33)]	28 (25) [21 (27)]	28 (23) [21 (27)]	30 (25) [21 (30)]	27 (22) [21 (28)]	24 (20) [19 (23)]	22 (21) [16 (20)]	22 (19) [15 (22)]	24 (23) [16 (25)]	20 (20) [13 (19)]	26	23%, 15–41%	< 0.001
CRP (mg/L)	21 (23) [12 (24)]	18 (27) [8 (19)]	16 (21) [7 (17)]	17 (24) [7 (16)]	16 (27) [6 (14)]	14 (20) [5 (13)]	12 (19) [5 (11)]	12 (21) [5 (11)]	14 (22) [5 (15)]	11 (16) [5 (10)]	15	19%, 11–37%	< 0.001
TJC28 (0–28)	9.3 (6.8) [8 (10)]	8.3 (7.0) [7 (9)]	7.3 (6.6) [5 (9)]	7.2 (6.4) [5 (8)]	6.7 (5.9) [5 (8)]	6.1 (6.3) [4 (8)]	4.6 (5.5) [2 (7)]	5.2 (5.5) [4 (7)]	5.7 (5.7) [4 (8)]	4.8 (5.8) [3 (7)]	6.5	15%, 7–37%	< 0.001
SJC28 (0–28)	7.3 (5.3) [6 (8)]	6.2 (5.1) [5 (6)]	5.7 (5.1) [4 (6)]	5.2 (4.7) [4 (5)]	4.4 (4.5) [3 (5)]	4.0 (4.7) [3 (5)]	3.1 (4.1) [2 (5)]	3.6 (4) [3 (6)]	3.7 (4.0) [3 (5)]	2.9 (4.1) [1 (4)]	4.6	15%, 7–37%	< 0.001
IGA (VAS, 0–100 mm)	45 (21)	40 (21)	38 (20)	40 (21)	37 (19)	37 (21)	27 (21)	32 (21)	31 (19)	28 (21)	36	34%, 22–50%	< 0.001
DAS28	5.3 (1.4)	5.0 (1.4)	4.8 (1.4)	4.7 (1.5)	4.6 (1.4)	4.3 (1.5)	3.8 (1.6)	4.0 (1.5)	4.2 (1.6)	3.8 (1.6)	4.4	29%, 18–47%	< 0.001
**Patient-Reported Outcome Measures**													
PGA (VAS, 0–100 mm)	62 (22)	58 (25)	58 (25)	56 (24)	55 (24)	51 (26)	47 (28)	49 (28)	55 (27)	49 (28)	54	16%, 9–35%	< 0.001
Pain (VAS, 0–100 mm)	57 (24)	54 (26)	54 (25)	53 (24)	51 (26)	48 (26)	45 (27)	47 (28)	52 (27)	47 (28)	51	23%, 13–52%	< 0.001
MHAQ (0–3)	1.0 (0.6)	0.9 (0.6)	0.9 (0.5)	0.8 (0.5)	0.8 (0.6)	0.6 (0.6)	0.7 (0.6)	0.7 (0.6)	0.8 (0.6)	0.7 (0.6)	0.8	20%, 12–38%	< 0.001
Fatigue (VAS, 0–100 mm)	58 (29)	55 (28)	57 (29)	53 (28)	56 (27)	51 (29)	48 (31)	52 (31)	54 (30)	51 (31)	54	38%, 15–56%	< 0.001
Morning Stiffness (hr)	2.1 (1.7)	1.9 (1.7)	1.9 (1.7)	1.7 (1.6)	1.7 (1.7)	1.5 (1.6)	1.5 (1.6)	1.6 (1.6)	1.6 (1.7)	1.4 (1.6)	1.7	40%, 16–59%	< 0.001

*Note*: Categorical variables are presented as percentages and continuous variables as mean with standard deviation (SD). Variables ESR, CRP, TJC28, and SJC28 also show median with interquartile range [Median (Interquartile Range)] below their Mean (SD). Missing data are presented as mean with range. χ^2^ test for categorical variables and one-way ANOVA for continuous variables was used to test for differences during follow-up of ten years

Occupation Status: Enabled Workers (< 65 years old) = Full Job, Part-time Job, Student, Maternity Leave, Paternity leave, Sick Leave, Unemployed, Early Retirement, Part-time job/Sick Leave, Part-time job/Unemployed), Disabled Workers (< 65 years) = Part-time Job/Disabled Early Retirement, Early Retirement due to Disability, Early Retirement due to RA, Medical Rehabilitation, Occupational Rehabilitation

*Abbreviations*: *RA* Rheumatoid Arthritis, *b/tsDMARDs* biologic and target synthetic Disease-Modifying Antirheumatic Drugs, *BMI* Body Mass Index, *CCP* Anti-cyclic citrullinated peptide, *RF* Rheumatoid Factor, *ESR* Erythrocyte Sedimentation Rate, *CRP* C-Reactive Protein, *TJC28* Tender 28-Joint Count, *SJC28* Swollen 28-Joint Count, *IGA* Investigators Global Assessment, *VAS* Visual Analog Scale (Measured 0–100), *DAS28* Disease Activity Score, *LDA* Low Disease Activity, *PGA* Patient Global assessment, *MHAQ* Modified Health Assessment Questionnaire

Both in naïve and non-naïve treatment groups, the disease activity level at the start of a new b/tsDMARD treatment decreased from 2010 to 2019. For naïve users, the mean DAS28 was 5.0 in 2010 and 3.8 in 2019, whereas DAS28 fell from 5.3 in 2010 to 3.8 in 2019 in the non-naïve group. A statistically significant difference was found for all PROM variables for non-naïve patients. However, in RA patients naïve to b/tsDMARDs, there were non-significant changes in VAS for pain and fatigue.

### Cost

The total treatment expenditure for b/tsDMARDs was lowest in 2010 (treating 1959 RA patients) with 25.6 million EUR, highest in 2014 (39.6 million EUR for treating 3448 patients), and second lowest in 2019 (28.9 million EUR for treating 4209 patients). Detailed information is shown in Table 4 for current users of b/tsDMARDs and the subgroups TNFi, non-TNFi, and tsDMARDs for the different 10 years. Table 4 also shows the numbers treated, the cost of b/tsDMARDs drugs started in the different years (for all and those naïve to b/tsDMARDs), and the subgroup TNFi non-TNFi and tsDMARDs.

**Table 4:** B/tsDMARDs treatment and cost in Norwegian RA outpatient clinic patients shown during 2010–2019

	2010	2011	2012	2013	2014	2015	2016	2017	2018	2019	Mean (Range) (2010–2019)
	(*n* = *4909*)	(*n* = *7256*)	(*n* = *7993*)	(*n* = *7278*)	(*n* = *8023*)	(*n* = *9057*)	(*n* = *9176*)	(*n* = *9225*)	(*n* = *9102*)	(*n* = *9335*)	
Number of treated patients with b/tsDMARDs and number of patients with double or multiple b/tsDMARDs registration errors (RE)											
b/tsDMARD users, N	1936	2855	3136	3060	3419	3688	3770	3869	3869	4158	
b/tsDMARD RE, N [%]	23 [1.2]	27 [0.9]	23 [0.7]	28 [0.9]	29 [0.8]	45 [1.2]	139 [3.7]	96 [2.5]	54 [1.4]	51 [1.2]	1.5% (0.7–3.7%)
Number of registered b/tsDMARDs **(**Percentage of n**) [**Percentage of the corresponding medication group**]**											
b/tsDMARDs (%)	1959 (40)	2882 (40)	3159 (40)	3088 (42)	3448 (43)	3733 (41)	3909 (43)	3965 (43)	3923 (43)	4209 (45)	42% (40–45%)
TNFi [%]	1485 [76]	2134 [74]	2319 [73]	2262 [73]	2469 [72]	2544 [68]	2696 [69]	2668 [67]	2462 [63]	2689 [64]	70% (64–76%)
Non-TNFi [%]	474 [24]	748 [26]	840 [27]	826 [27]	979 [28]	1189 [32]	1213 [31]	1262 [32]	1183 [30]	1084 [26]	28% (24–32%)
tsDMARD [%]	NA	NA	NA	NA	NA	NA	NA	35 [0.9]	278 [7.1]	436 [10.4]	6.1% (0.9–10%)
Number of b/tsDMARD users treated with csDMARDs, Methotrexate, and Glucocorticoids (percentage of b/tsDMARD users)											
csDMARD, N (%)	1430 (74)	2086 (73)	2256 (72)	2208 (72)	2415 (71)	2596 (70)	2630 (70)	2676 (69)	2568 (66)	2706 (65)	70% (65–74%)
Methotrexate, N (%)	1309 (68)	1885 (66)	2035 (65)	1983 (65)	2163 (63)	2282 (62)	2288 (61)	2327 (60)	2243 (58)	2357 (57)	62% (57–68%)
Glucocorticoids, N (%)	816 (42)	1123 (39)	1181 (38)	1136 (37)	1210 (35)	1277 (35)	1208 (32)	1189 (31)	1144 (30)	1199 (29)	35% (29–42%)
**Naïve Starting b**/**tsDMARD users vs. Non-Naïve starting b**/**tsDMARD users** (percentage of starting b/tsDMARD users) **[**Percentage of the corresponding medication group**]**											
All starting b/tsDMARDs	832	887	875	857	852	946	1671	1342	1068	1475	
Naïve to b/tsDMARDs	378 (45)	424 (48)	421 (48)	386 (45)	356 (42)	367 (39)	400 (24)	418 (31)	408 (38)	409 (28)	39% (24–48%)
Naïve to TNFi [%]	326 [86]	382 [90]	384 [91]	350 [91]	310 [87]	308 [84]	362 [91]	372 [89]	327 [80]	355 [87]	88% (80–91%)
Naïve to Non-TNFi [%]	52 [14]	42 [9.9]	37 [8.8]	36 [9.3]	46 [13]	59 [16]	38 [9.5]	46 [11]	51 [13]	23 [5.6]	11% (5.6–16%)
Naïve to tsDMARDs [%]	NA	NA	NA	NA	NA	NA	NA	NA	30 [7.4]	31 [7.6]	7.5% (7.4–7.6%)
Non-naïve to b/tsDMARDs	454 (55)	463 (52)	454 (52)	471 (55)	496 (58)	579 (61)	1271 (76)	924 (69)	660 (62)	1066 (72)	61% (52–76%)
TNFi [%]	213 [50]	252 [54]	282 [62]	281 [60]	278 [56]	364 [63]	1053 [83]	637 [69]	337 [51]	518 [49]	60% (49–83%)
Non-TNFi [%]	241 [53]	211 [46]	172 [38]	190 [40]	218 [44]	215 [37]	218 [17]	232 [25]	133 [20]	321 [30]	35% (17–53%)
tsDMARDs [%]	NA	NA	NA	NA	NA	NA	NA	55 [6.0]	190 [29]	227 [21]	19% (6.0–29%)
**The annual mean cost of b**/**tsDMARDs in thousand Euro [Adjusted consume price index price for 2010 NOK value]**											
Current b/tsDMARD cost	13.1 [13.1]	10.6 [10.5]	11.5 [11.1]	10.8 [10.3]	11.5 [10.7]	10.5 [9.7]	9.4 [8.3]	9.6 [8.4]	8.2 [7.1]	6.9 [5.8]	
Naïve b/tsDMARD cost	13.0 [13.0]	10.3 [10.2]	11.0 [10.6]	10.1 [9.6]	9.1 [8.4]	6.6 [6.1]	6.4 [5.7]	6.9 [6.0]	5.3 [4.6]	3.2 [2.7]	
Non-Naïve b/tsDMARD cost	12.9 [12.9]	10.9 [10.7]	11.7 [11.3]	10.8 [10.3]	10.5 [9.8]	8.1 [7.5]	7.6 [6.7]	7.6 [6.7]	5.9 [5.1]	4.6 [3.9]	
**The annual total cost of b**/**tsDMARDs in Million Euro [Adjusted consume price index price for 2010 NOK value]**											
Current b/tsDMARD cost	25.6 [25.6]	30.7 [30.3]	36.4 [35.0]	33.4 [31.7]	39.6 [36.8]	39.2 [36.1]	36.6 [32.5]	38.1 [33.3]	32.3 [28.0]	28.9 [24.4]	
Naïve b/tsDMARD cost	4.9 [4.9]	4.4 [4.3]	4.6 [4.5]	3.9 [3.7]	3.2 [3.0]	2.4 [2.2]	2.6 [2.3]	2.9 [2.5]	2.1 [1.9]	1.3 [1.1]	
Non-Naïve b/tsDMARD cost	5.9 [5.9]	5.0 [5.0]	5.3 [5.1]	5.1 [4.8]	5.2 [4.8]	4.7 [4.3]	9.6 [8.5]	7.1 [6.2]	3.9 [3.4]	4.9 [4.2]	

*Note*: Data are shown for current users b/tsDMARDs and patients starting a b/tsDMARDs both naïve and not naïve to previous use of b/tsDMARDs

Drugs included in TNFi: etanercept, etanercept SB4, infliximab, infliximab CT-P13, adalimumab, golimumab, certolizumab pegol. Drugs included in Non-TNFi: rituximab, rituximab GP2013, abatacept, and tocilizumab. Drugs included in tsDMARDs: baricitinib and tofacitinib

*Abbreviations*: *RA* Rheumatoid Arthritis, *b/tsDMARDs* biologic and target synthetic Disease-Modifying Antirheumatic Drugs, *tsDMARDs* target Synthetic DMARDs, *csDMARDs* conventional synthetic DMARDs, *TNFi* Tumor Necrosis Factor inhibitor (TNFi and Non-TNFi are subcategories of biologic DMARDs), *NA* Not available, *NOK* Norwegian Krones

The mean cost to treat a current RA user with b/tsDMARDs decreased by approximately 47% from 13.1 thousand EUR in 2010 to 6.9 thousand EUR in 2019 (Table 4). For both naïve and non-naïve b/tsDMARD users, the annual mean cost was markedly reduced from 2010 to 2019 by approximately 75 and 64% (13,0 thousand to 3.2 thousand and from 12.9 thousand to 4.6 thousand, respectively). Adjusted for CPI as displayed in Table 4, the reduction from 2010 to 2019 was even higher: for mean current users 56%, naïve users 80%, and non-naïve users 70%. When applying the tender results from 2020 on the 2019 population, the reduction was even higher with the estimated annual mean cost for current b/tsDMARDs users 5.8 thousand EUR and for naïve users 2.4 thousand EUR, which yields a cost reduction from 2010 of 56 and 82% and adjusted for CPI 64 and 85%, respectively.

**Fig. 1:**
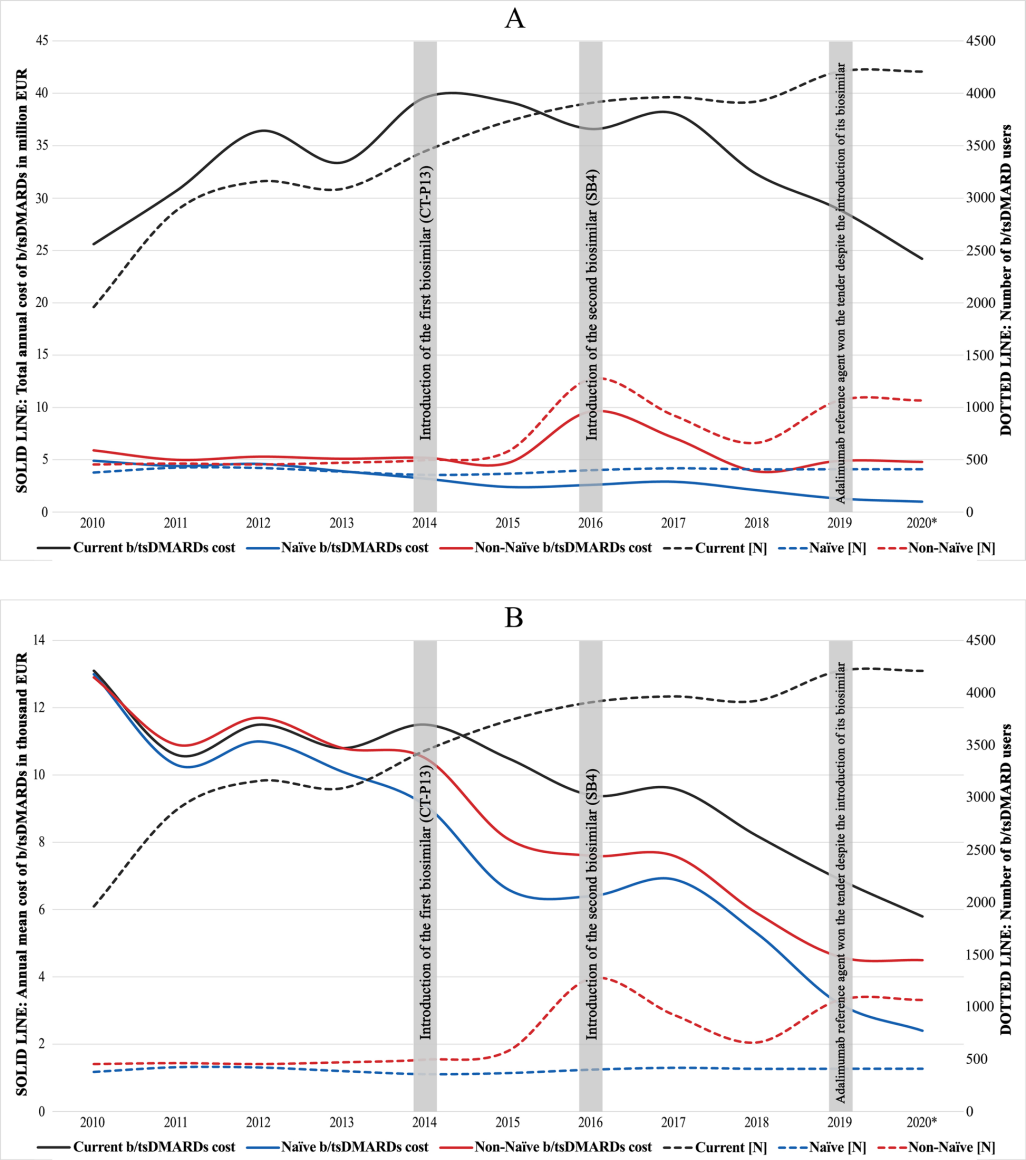
**A** and **B**: Number of Norwegian RA patients and treatment cost for current b/tsDMARDs users, those starting on a new b/tsDMARD for the first time (naïve), and those starting on a new b/tsDMARD not the first time (non-naïve). *Note*: In Fig. **A** the total cost is shown. Naïve = starting on a new b/tsDMARD for the first time, Non-Naïve = starting on a new b/tsDMARD not for the first time, 2020* = The 2020 tender results are applied in the 2019 population. *Abbreviations*: N = Number of patients with rheumatoid arthritis in the BioRheuma project, EUR = Euros, RA = Rheumatoid Arthritis, b/tsDMARDs = biologic and target synthetic Disease-Modifying Antirheumatic Drugs. *Note*: In Fig. **B** the mean cost to treat one patient is shown for the three groups. Naïve = starting on a new b/tsDMARD for the first time, Non-Naïve = starting on a new b/tsDMARD not for the first time, 2020* = The 2020 tender results are applied in the 2019 population. *Abbreviations*: N = Number of patients with rheumatoid arthritis in the BioRheuma project, EUR = Euros, RA = Rheumatoid Arthritis, b/tsDMARDs = biologic and target synthetic Disease-Modifying Antirheumatic Drugs

Figure 1A visualizes the change in total costs for treating RA patients with b/tsDMARDs for current users and for naïve and non-naïve starters of b/tsDMARDs and numbers of treated patients. Figure 1B shows the mean cost to treat one patient in the three groups.

### Completeness of patient recruitment

The estimated RA-prevalence based on BioRheuma data for each year and center is shown in a supplementary table (see Additional file 2). In 2019 the estimated overall prevalence (≥20 years old) was 0.3%, ranging at the single centers from 0.2 to 0.5%.

## Discussion

The main finding in this study is an estimated 47% reduction (56% CPI-adjusted) in the annual per-patient cost of b/tsDMARD from 2010 to 2019 in Norway. During this period, a national tender system for the prescription of b/tsDMARDs was implemented. The estimated annual cost reduction for naïve b/tsDMARD users was 75% (79.5% CPI-adjusted). Cost simulation using 2020 tender results on the 2019 population treatment data found that reduction increased further to 82% (85% CPI-adjusted) from 2010 for naïve patients.

The findings in our study suggest that the implemented tender system for b/tsDMARD procurements in Norway for the last 10 years may have facilitated positive competition between pharmaceutical companies and thus served as a market mechanism to reduce prices. The Norwegian Pharmaceutical Procurement Cooperation, a subdivision of the Norwegian Hospital Procurement Trust, has annually released lists of their recommendation for b/tsDMARDs use based on the results of the tender. The prescribing physicians are not obliged by law to follow the annual recommendations and may therefore choose another drug in case of individual reasons. However, the regional health trusts strongly advise and monitor the adherence to the annual (tender-based) recommendations. Since the original cost on specific b/tsDMARD is confidential, we can only report the total average cost of the assessed b/tsDMARDs. However, among the current b/tsDMARDs users, many patients are also using more expensive b/tsDMARDs on the tender list, which is reflected in the slower drop in prices shown in Table 4 and Fig. 1B.

The expiration of patents for reference bDMARDs has enabled the development and production of biosimilar bDMARDs, reaching the market at lower costs. In 2014 infliximab CT-P13 was the first biosimilar to reach the Norwegian market, followed by etanercept SB4 in 2016 [13, 14]. In 2016, a high increase was observed in prescription among RA patients who started on a b/tsDMARDs not being naïve to b/tsDMARDs compared to the steady rate years before. This is explained by the mandatory switching from reference agent to etanercept SB4, which in this study is defined as non-naïve starters on b/tsDMARDs.

In the 2019 Norwegian tender process, several companies manufacturing biosimilar adalimumab drugs gave price offers. However, the reference adalimumab won the tender by offering a lower price than what was offered for the biosimilars. The same was seen for etanercept in 2020, where the reference and not a biosimilar drug won. This shows that biosimilars influence the competition between pharmaceutical companies by influencing producers of reference bDMARDs to reduce their prices in order to win the tender. In 2020 however, the biosimilar GP2017 adalimumab won the tender process.

In Denmark, estimated accumulated price and quantitative data have been published for infliximab, etanercept, and adalimumab after the expiration of a patent [15, 16]. When the adalimumab biosimilar reached Denmark’s market in October 2018, the price for adalimumab dropped by 83% within 3 months. Whereas between September 2018 to September 2019, the use of adalimumab increased by approximately 35% [15].

The third mechanism used in Norway and Denmark to promote rapid cost reduction for bDMARDs is the recommended switch to the cheapest available substance when generics or biosimilars are available. In Norway, this switch has to be done by the treating rheumatologist and cannot be performed by the pharmacist, e.g., at the pharmacy.

As shown in our study, the impact of a tender system to reduce drug cost is a mechanism that may increase the availability of b/tsDMARDs to treat inflammatory arthritis, e.g., RA. This may be particularly important for low-income countries where RA patients have been shown to have higher disease activity than higher-income countries [5–7, 17, 18].

The previously documented improvement in clinical outcomes for RA patients in the new millennium in Norway [2, 3] and other countries [19–24] was also found in our study. Aga et al., in the NOR-DMARD multicenter study, found that remission rates in RA patients after 6 months of TNFi (and methotrexate) treatment had increased from 17% in the period 2000–2002 to 46% in the period 2009–2010 [3]. Disease duration before starting a TNFi had decreased from a median of 8.0 years (2000–2002) to 3.8 years (2009–2010) [3]. In comparison, in our study, the percentage of patients in DAS28 remission increased from 42% in 2010 to 67% in 2019, whereas disease duration in RA patients who started naïve on b/tsDMARDs did not change substantially.

Treatment with b/tsDMARDs in randomized clinical trials has been shown to improve occupational outcomes [25–27]. From the Swedish bDMARD registry, 35% of work-disabled RA patients with a disease duration of fewer than 5 years were found to regain their work ability within 3 years after starting a TNFi. With a disease duration of 5 years or more, the work recovery proportion was only 14% [28]. In our study, we did not see a significant change in the proportion of enabled workers across the 10 years. However, we saw a significant difference of roughly 10% (59% vs. 70%) among enabled workers when comparing those who were b/tsDMARD users vs. non-b/tsDMARD users (supplementary Table 1). Respectively, their average disease duration was 14 years vs. 9 years. When comparing the mean of naïve b/tsDMARDs users (Table 2) with non-naïve b/tsDMARDs users (Table 3) in the same manner, we observed 72% enabled workers with a six-year disease duration vs. 57% enabled workers with 12 years disease duration.

In the QUEST-RA study with data collected between 2005 and 2009 from 32 countries, 37% of previously work-enabled RA patients aged 65 years and younger reported occupational disability at the onset of RA symptoms (median observation period of 9 years) [29]. Despite the major differences in disease activity in their study, there was no significant difference in the proportion of work-enabled RA patients between countries with high and low gross domestic product (GDP). RA patients in low-GDP countries remained working despite high levels of disability and disease activity, suggesting that cultural and economic differences between societies also impact work disability rates in RA patients [29].

Our study’s major strength is that the data collected is standardized for all RA outpatients independent of treatment using the same hospital computer system. This is in contrast to some registry-based studies that either only included selected patient groups using b/tsDMARDs or patients who initiated treatment with csDMARDs and/or b/tsDMARDs (e.g., the Norwegian NOR-DMARD registry) [30]. Another strength is that the included patients come from 10 centers spread across Norway. Selection bias, if present, would most likely affect the first years of the 10-year period as the number of registered patients was lower than at the end of the period. However, no significant changes were seen between the RA patients for age, sex, CCP, and RF status.

Furthermore, comparing the estimated mean prevalence for RA of 0.3% in 2019 (single centers range 0.2 to 0.5%) in our study with a population-based prevalence of 0.4% in Oslo (1994) for the age group 20–80 years and 0.5% in Tromsø (1994) for the age group 20 years and older indicate a low grade of selection bias, at least in some centers [8, 9]. RA patients followed by privately practicing rheumatologists have not been included in the analysis and may partly explain lower prevalence estimates in some centers. However, we have reason to believe that both internal validity for each center and external validity for Norway are satisfactory.

The relatively high rate of missing data for disease activity measures is a limitation. Nevertheless, as argued above, we find this less likely to be caused by a systematic bias and is most likely based on random. Another limitation is the reduced effort of including patients in the BioRheuma projects during the early phase of the 10-year period. Therefore, the increasing percentage of included patients may be strongly affected by the examining physician’s interest in including the patient into the GoTreatIt Rheuma database. Also, it cannot be excluded that the improved disease outcome across the 10 years may have improved due to other factors such as earlier diagnosis, starting b/tsDMARDs at a lower disease activity, improved self-management, fewer comorbidities, and other aspects that may have reduced the patient global assessment (a key component of DAS28) besides the effect of b/tsDMARDs.

## Conclusions

In conclusion, our data shows that the average annual costs of treating a Norwegian RA patient with b/tsDMARD over the 10 year period 2010–19 were reduced by 47% for any user, and by 75% for naïve b/tsDMARD users. When adjusting for CPI, the percentage reduction was even higher. In Norway, with a tax-based healthcare system, we show that treatment with b/tsDMARDs has become more available at a lower cost, and the threshold for starting b/tsDMARDs has decreased significantly. Although not confirming causality, there is strong reason to believe that the national tender system has contributed significantly to this favorable price reduction for b/tsDMARDs in Norway.

## Supplementary Information

**Additional file 1: Supplementary Table.** Aggregated data for demographic, disease outcome, and treatment during 2010–2019.

**Additional file 2: Supplementary Table.** RA prevalence of BioRheuma registered patients (≥20 years) shown for all participating centers.

## Data Availability

Data are available on reasonable request and must be approved by all participating centers. Please contact the corresponding author by email to request the data from this study.

## Abbreviations

ANOVA: Analysis of variance
BioRheuma: BIOlogic treatment of patients suffering from inflammatory RHEUMAtic disorders in Norway
BMI: Body mass index
b/tsDMARDs: Biologic and targeted synthetic disease-modifying antirheumatic drugs
CPI: Consumer price index
CRP: C-reactive protein
csDMARDs: Conventional synthetic DMARDs
ESR: Erythrocyte sedimentation rate
EUR: Euros
GDP: Gross domestic product
IGA: Investigator global assessment
MHAQ: Modified Health Assessment Questionnaire
NOK: Norwegian Kroners
PGA: Patient global assessment
PROMs: Patient-reported outcome measures
RA: Rheumatoid arthritis
REC: Regional ethical committee
RF: Rheumatoid factor
SJC28: 28 swollen joint count
SPSS: Statistical package for social sciences
TJC28: 28 tender joint count
SD: Standard deviation
TNFi: Tumor Necrosis Factor inhibitors
VAS: Visual analog scale
DAS28: Composite 28 joint count Disease Activity Score
