# Supplementary material for: Exploring drug cost and disease outcome in rheumatoid arthritis patients treated with biologic and targeted synthetic DMARDs in Norway in 2010–2019 – a country with a national tender system for prescription of costly drugs
Source: BMC Health Serv Res. 2022 Jan 10;22:48. doi: 10.1186/s12913-021-07425-w (PMC8743354; doi:10.1186/s12913-021-07425-w)
Supplement: Supplementary file 1 — Additional file 1: Supplementary Table. Aggregated data for demographic, disease outcome, and treatment during 2010–2019. [file 12913_2021_7425_MOESM1_ESM.doc]

**Supplementary table: Aggregated data for demographic, disease outcome, and treatment during 2010-2019.**

|  | **All BioRheuma patients (b**/**tsDMARDs, csDMARDs, Glucocorticoids); 2010-2019** | | | |  | **b**/**tsDMARDs treated patients; 2010-2019** | | | |  | **Non-b**/**tsDMARDs treated patients**  **(2010-2019)** | | | |  |  |
| --- | --- | --- | --- | --- | --- | --- | --- | --- | --- | --- | --- | --- | --- | --- | --- | --- |
|  | Mean | Range | Missing Data  Mean, Range | P-value |  | Mean | Range | Missing Data  Mean, Range | P-value |  | Mean | Range | Missing Data  Mean, Range | P-value |  | P-value* |
| **Demographics** | | | | | | | | | | | | | | | | |
| Age (Years) | 61.4 | 60.7-62.4 | 1%, 0-10% | <0.001 |  | 59.3 | 58.7-59.8 | 1.1%, 0-11% | 0.044 |  | 62.8 | 62.1-64.2 | 0.9%, 0-9% | <0.001 |  | <0.001 |
| Female | 70.6% | 71.0-71.8% | 1%, 0-10% | <0.001 |  | 72.6% | 71.7-73.5% | 1.1%, 0-11% | 0.938 |  | 70.4% | 70.0-70.6% | 0.9%, 0-9% | 0.997 |  | <0.001 |
| BMI (kg/m^2^) | 26.0 | 25.7-26.3 | 12%, 3-49% | <0.001 |  | 25.9 | 25.5-26.3 | 9.9%, 2-57% | <0.001 |  | 26.0 | 25.7-26.3 | 13.5%, 4-44% | <0.001 |  | 0.022 |
| Education (Years) | 12.0 | 11.6-12.4 | 11.7%, 2-46% | <0.001 |  | 12.3 | 11.9-12.7 | 9.3%, 1-55% | <0.001 |  | 11.7 | 11.4-12.1 | 13.4%, 3-41% | <0.001 |  | <0.001 |
| Current Smokers | 18.4% | 14.1-23.7% | 10.2%, 2-41% | <0.001 |  | 17.7% | 13.7-22.6% | 8.2%, 1-50% | <0.001 |  | 18.9% | 14.5-24.2% | 11.5%, 3-35% | <0.001 |  | <0.001 |
| Disease Duration (Years) | 10.9 | 10.5-11.5 | 0.0%, 0-0% | <0.001 |  | 14.0 | 12.9-14.6 | 1.1%, 0-11% | <0.001 |  | 8.9 | 8.6-9.0 | 0.0%, 0-0% | 0.348 |  | <0.001 |
| Enabled Workers (<65 years) | 64.6% | 62.6-68.9% | 9.1%, 2-37% | 0.001 |  | 59.1% | 56.6-62.7% | 7.4%, 1-45% | 0.393 |  | 69.6% | 68.2-73.2% | 10.5%, 3-31% | 0.055 |  | <0.001 |
| **Biomarkers** | | | | | | | | | | | | | | | | |
| CCP Positive | 74.8% | 74.1-75.5% | 31.4%, 19-47% | 0.808 |  | 80.9% | 80.1-82.0% | 27.0%, 14-42% | 0.900 |  | 70.0% | 68.8-70.9% | 34.5%, 22-49% | 0.813 |  | <0.001 |
| RF Positive | 67.4% | 66.7-68.9% | 45.4%, 32-59% | 0.701 |  | 73.1% | 72.1-74.7% | 44.7%, 29-61% | 0.798 |  | 63.4% | 62.2-65.4% | 46.0%, 36-58% | 0.705 |  | <0.001 |
| **Disease Activity** | | | | | | | | | | | | | | | | |
| ESR (mm/h) | 16.4 | 14.6-19.0 | 30.6%, 26-38% | <0.001 |  | 15.5 | 13.5-18.6 | 26.4%, 20-32% | <0.001 |  | 17.1 | 15.5-19.1 | 33.6%, 28-43% | <0.001 |  | <0.001 |
| CRP (mg/L) | 7.3 | 6.6-8.7 | 21.7%, 19-26% | <0.001 |  | 6.6 | 6.0-8.4 | 18.8%, 15-27% | <0.001 |  | 7.9 | 7.2-8.9 | 23.8%, 21-29% | <0.001 |  | <0.001 |
| TJC28 (0-28) | 2.4 | 1.7-3.3 | 15.9%, 12-20% | <0.001 |  | 2.4 | 1.7-3.4 | 14.5%, 9-19% | <0.001 |  | 2.3 | 1.7-3.3 | 16.8%, 14-21% | <0.001 |  | 0.728 |
| SJC28 (0-28) | 1.4 | 0.9-2.2 | 15.9%, 12-20% | <0.001 |  | 1.4 | 0.8-2.3 | 14.5%, 9-19% | <0.001 |  | 1.5 | 1.0-2.2 | 16.8%, 14-21% | <0.001 |  | <0.001 |
| IGA (VAS, 0-100 mm) | 14.7 | 11.9-17.1 | 38.6%, 31-51% | <0.001 |  | 15.2 | 12.0-18.1 | 40.2%, 35-50% | <0.001 |  | 14.4 | 11.8-16.6 | 37.6%, 24-52% | <0.001 |  | <0.001 |
| DAS28 | 2.7 | 2.5-3.1 | 33.7%, 30-39% | <0.001 |  | 2.7 | 2.4-3.1 | 30.4%, 26-34% | <0.001 |  | 2.7 | 2.5-3.1 | 36.0%, 33-42% | <0.001 |  | <0.001 |
| DAS28 Remission | 55.6% | 41.0-65.4% | 33.7%, 30-39% | <0.001 |  | 56.4% | 41.6-66.8% | 30.4%, 26-34% | <0.001 |  | 54.9% | 40.6-64.2% | 36.0%, 33-42% | <0.001 |  | <0.001 |
| DAS28 LDA | 16.2% | 14.1-18.8% | 33.7%, 30-39% | <0.001 |  | 16.3% | 14.1-18.4% | 30.4%, 26-34% | 0.034 |  | 16.2% | 14.0-19.0% | 36.0%, 33-42% | <0.001 |  | 0.991 |
| **Patient-Reported Outcome Measures** | | | | | | | | | | | | | | | | |
| PGA (VAS, 0-100 mm) | 33.3 | 32.3-35.8 | 11.2%, 9-12% | <0.001 |  | 32.5 | 31.9-33.3 | 10.0%, 8-11% | 0.270 |  | 33.9 | 32.7-37.4 | 12.0%, 10-13% | <0.00 |  | <0.001 |
| Pain (VAS, 0-100 mm) | 33.5 | 32.6-35.1 | 21%, 14-45% | <0.001 |  | 32.5 | 31.7-33.5 | 20.4%, 14-53% | 0.204 |  | 34.1 | 32.9-36.4 | 21.5%, 14-40% | <0.00 |  | <0.001 |
| MHAQ (0-3) | 0.45 | 0.43-0.49 | 14.2%, 12-23% | <0.001 |  | 0.47 | 0.45-0.51 | 14.9%, 10-37% | 0.001 |  | 0.42 | 0.40-0.48 | 13.7%, 12-16% | <0.00 |  | <0.001 |
| Fatigue (VAS, 0-100 mm) | 37.6 | 36.5-38.7 | 33.9%, 16-52% | 0.001 |  | 38.5 | 37.1-40.1 | 37.1%, 15-54% | 0.006 |  | 37.1 | 35.8-38.4 | 31.7%, 14-51% | 0.023 |  | <0.001 |
| Morning Stiffness (hr) | 0.9 | 0.8-0.9 | 37.4%, 16-56% | 0.092 |  | 0.9 | 0.8-0.9 | 41.7%, 16-59% | 0.614 |  | 0.9 | 0.8-0.9 | 34.5%, 15-53% | 0.179 |  | 0.528 |
| **Supplementary Treatment Overview** | | | | | | | | | | | | | | | | |
| csDMARD users, N (%) | 73.7% | 70.9-75.6% | 0%, 0-0 % | <0.001 |  | 70.2% | 65.1-73.9% | 0.0%, 0-0 % | <0.001 |  | 76.3% | 70.3-81.3% | 0.0%, 0-0 % | <0.001 |  | <0.001 |
| Methotrexate users, N (%) | 64.5% | 62.4-65.8% | 0%, 0-0 % | <0.001 |  | 62.4% | 56.7-67.6% | 0.0% ,0-0 % | <0.001 |  | 66.2% | 60.7-71.4% | 0.0%, 0-0 % | <0.001 |  | <0.001 |
| Glucocorticoid users, N (%) | 35.6% | 30.1-42.7% | 0%, 0-0 % | <0.001 |  | 34.7% | 28.8-42.1% | 0.0%, 0-0 % | <0.001 |  | 36.3% | 31.1-43.1% | 0.0%, 0-0 % | <0.001 |  | <0.001 |

*Note*: The table includes all RA patients registered in the BioRheuma at the participating centers, b/tsDMARDs treated patients, and non-b/tsDMARDs treated patients during 2010-2019. Categorical variables are presented as a percentage and continuous variables as mean with range. Missing data presented as mean and range. χ^2^ test for categorical variables and one-way ANOVA for continuous variables was used to test for differences during follow-up of ten years. *Abbreviation*: **RA** = Rheumatoid arthritis, **b**/**tsDMARDs** = biologic and target synthetic Disease-Modifying Antirheumatic Drugs, **BMI** = Body Mass Index, **CCP** = Anti-cyclic citrullinated peptide, **RF** = Rheumatoid Factor, **ESR** = Erythrocyte Sedimentation Rate, **CRP** = C-Reactive Protein, **TJC28** = Tender 28-Joint Count, **SJC28** = Swollen 28-Joint Count, **IGA** = Investigators Global Assessment, **VAS =** Visual Analog Scale (Measured 0-100), **DAS28** = Disease Activity Score, **LDA** = Low Disease Activity, **PGA** = Patient Global assessment, **MHAQ** = Modified Health Assessment Questionnaire, **csDMARDs** = conventional synthetic Disease-Modifying Antirheumatic Drugs. Occupation Status: **Enabled Workers** (<65 years old) = Full Job, Part-time Job, Student, Maternity Leave, Paternity leave, Sick Leave, Unemployed, Early Retirement, Part-time job/Sick Leave, Part-time job/Unemployed), Disabled Workers (< 65 years) = Part-time Job/Disabled Early Retirement, Early Retirement due to Disability, Early Retirement due to RA, Medical Rehabilitation, Occupational Rehabilitation. ***** = Shows the p-value between the mean of b/tsDMARDs treated patients (2010-2019) and non-b/tsDMARDs treated patients (2010-2019).
