# Supplementary material for: Exploring drug cost and disease outcome in rheumatoid arthritis patients treated with biologic and targeted synthetic DMARDs in Norway in 2010–2019 – a country with a national tender system for prescription of costly drugs
Source: BMC Health Serv Res. 2022 Jan 10;22:48. doi: 10.1186/s12913-021-07425-w (PMC8743354; doi:10.1186/s12913-021-07425-w)
Supplement: Supplementary file 2 — Additional file 2: Supplementary Table. RA prevalence of BioRheuma registered patients (≥20 years) shown for all participating centers. [file 12913_2021_7425_MOESM2_ESM.doc]

**Supplementary table**: RA prevalence of BioRheuma registered patients (≥20 years) shown for all participating centers.

|  | **2010** | **2011** | **2012** | **2013** | **2014** | **2015** | **2016** | **2017** | **2018** | **2019** | **Mean (Range)** |
| --- | --- | --- | --- | --- | --- | --- | --- | --- | --- | --- | --- |
| All Centers  (Range)  [N/SA] | 0.24%  (0.11-0.54%)  [4696/1.94^M^] | 0.31%  (0.15-0.54%)  [7205/2.36 ^M^] | 0.33%  (0.21-0.54%)  [7810/2.40^M^] | 0.32%  (0.21-0.51%)  [6849/2.14^M^] | 0.33%  (0.20-0.51%)  [7243/2.18^M^] | 0.30%  (0.20-0.49%)  [9044/2.99^M^] | 0.29%  (0.21-0.45%)  [9166/3.11^M^] | 0.29%  (0.20-0.45%)  [9213/3.14^M^] | 0.29%  (0.19-0.45%)  [9092/3.18^M^] | 0.29%  (0.20-0.46%)  [9323/3.21^M^] | 0.30%  (0.24-0.33%) |
| **Individual Centers** | | | | | | | | | | | |
| UNN [N/SA] | 0.30% | 0.40% | 0.45% | 0.49% | 0.42% | 0.45% | 0.43% | 0.40% | 0.37% | 0.38% | 0.41%  (0.30-0.49%) |
|  | [348/116165] | [465/117408] | [536/118850] | [596/120936] | [519/122724] | [562/124571] | [544/125752] | [503/127085] | [481/128272] | [487/129427] |  |
| STO  [N/SA] | 0.27% | 0.34% | 0.34% | 0.37% | 0.41% | 0.34% | 0.30% | 0.30% | 0.27% | 0.27% | 0.32%  (0.27-0.41%) |
|  | [581/217240] | [743/220443] | [768/224504] | [838/228995] | [959/232344] | [810/236020] | [715/239088] | [720/242669] | [669/245920] | [672/249670] |  |
| Førde  [N/SA] | * | 0.24% | 0.30% | 0.33% | 0.37% | * | 0.45% | 0.45% | 0.45% | 0.46% | 0.38%  (0.24-0.46%) |
|  |  | [183/77328] | [230/77947] | [260/78630] | [289/79142] |  | [359/80115] | [360/80851] | [365/81098] | [370/81022] |  |
| HUS  [N/SA] | 0.12% | 0.35% | 0.37% | 0.37% | 0.38% | 0.40% | 0.38% | 0.35% | 0.37% | 0.34% | 0.34%  (0.12-0.40%) |
|  | [381/ 310279] | [1110/316175] | [1187/321659] | [1222/327905] | [1278/334155] | [1370/339543] | [1307/344043] | [1228/347150] | [1307/349990] | [1191/352635] |  |
| Haugesund [N/SA] | 0.11% | 0.15% | 0.21% | 0.23% | 0.28% | 0.28% | 0.27% | 0.26% | 0.23% | 0.23% | 0.23%  (0.11-0.28%) |
|  | [381/350860] | [545/358166] | [771/364895] | [840/373236] | [1052/380021] | [1096/386638] | [1053/390468] | [1020/392742] | [926/394673] | [929/397717] |  |
| SSHF  [N/SA] | 0.54% | 0.54% | 0.54% | 0.51% | 0.51% | 0.49% | 0.41% | 0.42% | 0.36% | 0.38% | 0.47%  (0.36-0.54%) |
|  | [1097/203926] | [1123/207093] | [1130/210197] | [1091/213187] | [1107/215997] | [1066/219319] | [906/222085] | [951/224221] | [825/227349] | [870/229252] |  |
| Betanien  [N/SA] | * | 0.33% | 0.34% | 0.32% | 0.34% | 0.28% | 0.26% | 0.30% | 0.33% | 0.32% | 0.31%  (0.26-0.34%) |
|  |  | [1011/303902] | [1055/307582] | [1000/311026] | [1061/313848] | [887/316642] | [831/319921] | [960/322895] | [1062/325476] | [1052/327955] |  |
| MHH  [N/SA] | * | * | * | * | * | 0.21% | 0.21% | 0.20% | 0.19% | 0.20% | 0.22%  (0.19-0.21%) |
|  |  |  |  |  |  | [1180/562221] | [1228/572096] | [1157/582441] | [1132/592542] | [1213/602860] |  |
| DS  [N/SA] | 0.22% | 0.22% | 0.22% | 0.21% | 0.20% | 0.20% | 0.22% | 0.24% | 0.25% | 0.27% | 0.23%  (0.20-0.27%) |
|  | [1006/457327] | [1033/467318] | [1073/479214] | [1002/487975] | [978/496866] | [1033/507474] | [1115/516365] | [1230/523007] | [1342/528676] | [1471/535980] |  |
| LHR  [N/SA] | 0.31% | 0.34% | 0.36% | * | * | 0.35% | 0.37% | 0.36% | 0.32% | 0.35% | 0.35%  (0.31-0.37%) |
|  | [902/286619] | [992/288855] | [1060/291644] |  |  | [1040/297656] | [1108/298896] | [1084/300722] | [983/302508] | [1068/303694] |  |
| Patient (≥20) from the BioRheuma Project | | | | | | | | | | | |
|  | 95.7% | 99.3% | 97.7% | 94.1% | 90.3% | 99.9% | 99.9% | 99.9% | 99.9% | 99.9% |  |
| Norway's national population (≥20) | | | | | | | | | | | |
|  | 3618442 | 3674972 | 3737305 | 3797822 | 3852406 | 3906903 | 3953206 | 3995587 | 4034726 | 4072755 |  |
| SA coverage of BioRheuma centers in Norway | | | | | | | | | | | |
|  | 53.6% | 64.1% | 64.1% | 56.4% | 56.5% | 76.5% | 78.6% | 78.7% | 78.7% | 78.8% | 68.6% |

*Note*: The prevalence is estimated using BioRheuma age group ≥20 years and RA service area from the corresponding centers for the same age group. The prevalence that did not reach 0.1 was excluded from the further assessment due to a very low number (presented with *). Only prevalence above the cut-off value of 0.1 was analyzed for the mean across the ten years for each area, and to calculate prevalence for all hospitals and the total service area coverage. *Abbreviation*: **RA** = Rheumatoid Arthritis, **N** = Number of patients with rheumatoid arthritis in the BioRheuma project from individual centers, **SA** = Service Area (for rheumatoid arthritis patients) **UNN** = University Hospital of North Norway, **STO** = St. Olav's university hospital, **HUS** = Haukeland University Hospital, **SSHF** = Hospital of Southern Norway, **MHH** = Martina Hansen Hospital, **DS** = Diakonhjemmet Hospital, **LHR** = Lillehammer Hospital for Rheumatic Diseases, **^M^** = Millions.
